# Allergic Airway Disease in Mice Alters T and B Cell Responses during an Acute Respiratory Poxvirus Infection

**DOI:** 10.1371/journal.pone.0062222

**Published:** 2013-04-19

**Authors:** Crystal C. Walline, Sarita Sehra, Amanda J. Fisher, Lynette M. Guindon, Ian M. Kratzke, Jessica B. Montgomery, Kelsey P. Lipking, Nicole L. Glosson, Heather L. Benson, George E. Sandusky, David S. Wilkes, Randy R. Brutkiewicz, Mark H. Kaplan, Janice S. Blum

**Affiliations:** 1 Department of Microbiology & Immunology, Indiana University School of Medicine, Indianapolis, Indiana, United States of America; 2 Department of Pediatrics, HB Wells Center for Pediatric Research, Indiana University School of Medicine, Indianapolis, Indiana, United States of America; 3 Center for Immunobiology, Indiana University School of Medicine, Indianapolis, Indiana, United States of America; 4 Department of Pathology & Laboratory Medicine, Indiana University School of Medicine, Indianapolis, Indiana, United States of America; University of Iowa, United States of America

## Abstract

Pulmonary viral infections can exacerbate or trigger the development of allergic airway diseases via multiple mechanisms depending upon the infectious agent. Respiratory vaccinia virus transmission is well established, yet the effects of allergic airway disease on the host response to intra-pulmonary vaccinia virus infection remain poorly defined. As shown here BALB/c mice with preexisting airway disease infected with vaccinia virus developed more severe pulmonary inflammation, higher lung virus titers and greater weight loss compared with mice inoculated with virus alone. This enhanced viremia was observed despite increased pulmonary recruitment of CD8^+^ T effectors, greater IFNγ production in the lung, and high serum levels of anti-viral antibodies. Notably, flow cytometric analyses of lung CD8^+^ T cells revealed a shift in the hierarchy of immunodominant viral epitopes in virus inoculated mice with allergic airway disease compared to mice treated with virus only. Pulmonary IL-10 production by T cells and antigen presenting cells was detected following virus inoculation of animals and increased dramatically in allergic mice exposed to virus. IL-10 modulation of host responses to this respiratory virus infection was greatly influenced by the localized pulmonary microenvironment. Thus, blocking IL-10 signaling in virus-infected mice with allergic airway disease enhanced pulmonary CD4^+^ T cell production of IFNγ and increased serum anti-viral IgG1 levels. In contrast, pulmonary IFNγ and virus-specific IgG1 levels were reduced in vaccinia virus-treated mice with IL-10 receptor blockade. These observations demonstrate that pre-existing allergic lung disease alters the quality and magnitude of immune responses to respiratory poxviruses through an IL-10-dependent mechanism.

## Introduction

Respiratory viral infections such as rhinovirus, respiratory syncytial virus (RSV) and influenza are known to exacerbate or trigger the development of allergic airway disease (AAD) [1], [2]. Murine models of experimentally induced AAD have shown that allergic airway inflammation increases susceptibility to respiratory viruses, resulting in enhanced inflammation and alterations in host immune responses [3]–[7]. Infectious poxvirus transmission has been linked to endemic viruses as well as inadvertent exposure to vaccine strains such as vaccinia virus (VV) particularly in individuals with immune deficiencies or allergic diseases [8], [9]. Respiratory transmission of poxviruses such as VV can have severe consequences which have been attributed in part to the immunomodulatory properties of these viruses [10].

How the allergic microenvironment and cytokine-mediators within the atopic lung alter host responses and immunity to poxviruses remains poorly defined. AAD is often characterized by Th2-driven immune responses, eosinophilia, and airway hyperresponsiveness. The Th2 cytokines IL-4, IL-5 and IL-13 are associated with AAD, although IL-17 also contributes in some models [11]. Yet, induction of Th1 cytokines such as IFNγ and IL-12 abrogates Th2-induced inflammation in AAD [12]. Intranasal inoculation of poxviruses, such as VV, promotes high levels of the Th1 cytokine IFNγ and measurable IL-17 and IL-10 production in the lungs of mice [13]–[15]. T cells producing IFNγ and IL-17 can play protective roles in host responses to VV [14], [16]. Yet the biological importance and cellular sources of IL-10 in respiratory poxvirus infections, including those within the atopic lung, remain unclear. IL-10 can serve as an immunosuppressive cytokine to negatively regulate innate and adaptive immune responses late in parasitic, bacterial and viral infections [17], promoting chronic viral infections in some cases by limiting anti-viral immunity [18]–[20]. During respiratory cowpox inoculation in mice deficient in IL-10, pulmonary infiltrates increased but failed to enhance virus clearance [15]. The phenotype and function of these infiltrating cells were not examined. Whether changes in the atopic lung would similarly influence VV clearance or the role of IL-10 in pulmonary immunity to respiratory poxviruses, has yet to be tested.

To address whether preexisting allergic inflammation influences host responses to respiratory VV infection, a murine model of AAD was used. Here, BALB/c mice with AAD upon infection with VV developed more severe weight loss, peribronchiolar inflammation, and had diminished viral clearance compared to mice exposed to VV alone. Analysis of CD8^+^ T cells from virus-infected mice with AAD revealed differences in the hierarchy of responses to MHC class I–restricted viral epitopes in comparison with mice treated with virus alone. Virus-induced T cell cytokine and serum antibody production was differentially modulated by IL-10 signaling dependent upon allergic conditions. These results suggest that IL-10 may be important for controlling dysregulated T cells and modulating humoral responses during VV infection.

## Materials and Methods

### Induction of Allergic Airway Inflammation, VV Infection and Ab Treatment

The Indiana University School of Medicine Institutional Animal Care and Use Committee (#A4091), which is based at Indiana University and Purdue University at Indianapolis, has approved the animal use protocols for this study and complies with the guidelines set forth in the Animal Welfare Act, the National Research Council's Guide for the Care and Use of Laboratory Animals, and the Public Health Service's Policy on Humane Care and Use of Laboratory Animals. Intranasal treatments were administered under isoflurane sedation and mice were anesthetized with ketamine/xylazine prior to sacrifice. Mice were euthanized if weight loss exceeded 30% of original body weight and all efforts were made to minimize suffering. Eight to 10 week old BALB/c mice (Harlan Laboratories) were sensitized by i.p. injections of OVA/alum (20 µg OVA/2 mg alum, Sigma-Aldrich) on days 0 and 7. On day 14, mice were exposed to intranasal (i.n.) OVA (100 µg) per day for 6 consecutive days. Two days after the last i.n. challenge, mice were intratracheally (i.t.) administered 10^4^ PFU Western Reserve VV or mock treated with PBS. In some experiments, mice were administered blocking IL-10R-specific mAb or rat IgG1 isotype control mAb on days 3 and 6 (1 mg i.p. in 250 µl), and day 4 (0.15 mg i.n. in 20 µl) post-infection (Bio-Express). Mice were anesthetized, bled, tracheas were cannulated and lungs lavaged three times with 1.5 ml PBS to obtain bronchoalveolar lavage (BAL) fluid prior to tissue harvesting. BAL fluid and blood were centrifuged briefly to collect BAL cells/supernatant and serum, respectively.

### Unrestrained Whole-body Plethysmography

Non-invasive unrestrained whole-body plethysmography (Buxco Systems) was used to record altered patterns of breathing in mice as enhanced pause (Penh) in response to methacholine challenge at 7 days post infection (dpi). The non-invasive method allowed an evaluation of the lung function for the same cohort of mice that was used for ex vivo analyses at later time points.

### Lung Histological and Mucus Production Analyses

Murine lung tissue was fixed in 10% formalin and paraffin-embedded sections were stained with hematoxylin and eosin (H&E) to evaluate peribronchiolar and perivascular inflammation or periodic acid Schiff (PAS) to assess mucus production. PAS-stained tissue was also counterstained with hematoxylin to identify inflammatory cells. The inflammation (H&E) was scored in a blinded manner using light microscopy and a semi-quantitative scoring scale: 0, no inflammation; 1, minimal inflammation of peribronchiolar and periarterial tissues; 2, inflammation of peribronchiolar and periarterial tissues that extends around the entire vessel; 3, extensive inflammation of peribronchiolar and periarterial tissues that extends around the entire vessel and locally into parenchymal spaces; 4, severe inflammation of peribronchiolar, periarterial, and parenchymal spaces. The Aperio Scan Scope CS system was used for whole slide digital imaging of PAS slides. Computer-assisted morphometric analysis of digital images was done using the Aperio software and a modified positive pixel algorithm to distinguish between blue airway epithelial cells and red goblet cells. Additionally, digital images (20× magnification) were scored for epithelial disruption, goblet cell hyperplasia, giant cell pneumonia and perivascular lymphoid hyperplasia in a blinded manner using a semi-quantitative scale: 0, normal; 1, mild; 2, moderate; 3, severe.

### Virus Assays

VV stocks were sucrose gradient-purified. Titers for purified virus or lung tissue were determined by a standard viral plaque assay [21].

### Quantitative Real-time PCR (qRT-PCR)

Lung samples were preserved in RNAlater and total RNA extracted (RNeasy Mini kit, Qiagen). cDNA was generated using the High-Capacity cDNA Reverse Transcription Kit; qRT-PCR was performed using commercially available TaqMan primers and the ABI Prism 7500 Fast RT-PCR System (Applied Biosystems). Gene expression was quantitated relative to beta 2 microglobulin mRNA levels and presented as an arbitrary fold change compared with control samples.

### Lung Cell Isolation and Flow Cytometry

The right lung from each animal was homogenized using the gentleMACS Dissociator (Miltenyi) followed by Percoll gradient purification. For detection of intracellular cytokines, mononuclear cells were restimulated with 500 ng/ml ionomycin, and 50 ng/ml PMA in the presence of 1 µg/ml Brefeldin A for 5 h. Cells were stained at 4°C with 2.4G2 FcR blocking Ab present. APC subsets were detected using FL-1 autofluorescence, F4/80 APC-Cy7 (Biolegend), MHC class II PE, PDCA-1 Alexa Fluor 647 (eBioscience), CD11b PerCP-Cy5.5, CD11c PE-Cy7, and B220 PE-Cy5 (BD Biosciences). Lymphocytes were gated using scatter properties and T cell subsets were detected using CD4 PerCP-Cy5.5, CD8 APC-Cy7 (BD Biosciences) and then fixed and permeabilized (Foxp3 Fixation/Permeabilization kit, eBioscience). The cells were subsequently stained for intracellular IL-10 PE, IFNγ PE-Cy7 and Foxp3 APC (eBioscience). For epitope-specific tetramer staining, cells were stained with 2 µg/ml VV-specific MHC class I tetramers (NIH Tetramer Core Facility) and CD8 APC-Cy7, CD11c FITC, B220 FITC, F4/80 FITC and CD4 FITC (eBioscience) at 37°C during the final 30 m of restimulation (described above). Cells were stained with Live/Dead Fixable Green Dead Cell Stain Kit, 488 nm (Molecular Probes), fixed/permeabilized and stained for IFNγ and IL-10 as described above. CD8^+^ T cells were gated by excluding FITC^+^ non-CD8 cells and FITC^+^ dead cells (dump gate) and then selecting positively stained CD8^+^ cells in the FSC/SSC lymphocyte gate [22]. VV-specific biotinylated MHC class I monomers loaded with epitopes A52_75–83_ (K^d^), F2_26–34_ (L^d^), and E3_140–148_ (D^d^) were tetramerized in advance with streptavidin (SA)-allophycocyanin (Molecular Probes).

### ELISAs

ELISAs were performed using Costar EIA/RIA flat-bottomed 96-well plates coated over night with coating Ag diluted in 0.1 M carbonate buffer, pH 9.5. All antibodies and mouse serum were diluted in PBS/1% BSA. Cytokines were measured in BAL fluid using a standard ELISA with 2 µg/ml purified and 1 µg/ml biotinylated antibodies (BD Biosciences) and SA-HRP (0.2 µg/ml, Thermo Fisher). To determine levels of OVA-specific IgE, plates were coated with OVA (50 µg/ml, Sigma), blocked, and then diluted mouse serum (1∶30) was added and incubated overnight. Biotinylated anti-mouse IgE (1∶500, BD Biosciences) and SA-HRP (0.25 µg/ml) were used for detection. Anti-OVA serum IgE levels were reported as optical density (OD) at 405 nm. To determine levels of serum anti-VV Ig, plates were coated with VV (250 PFU/ml), blocked, and then dilutions of mouse serum were added and incubated overnight. Virus bound anti-VV antibodies of different isotypes were detected using anti-mouse IgG1-HRP (1∶3000, Southern Biotech), anti-mouse IgG2a-HRP (1∶1000, BD Biosciences) or anti-mouse IgM-biotin (1∶500, BD Biosciences) followed by SA-HRP (0.25 µg/ml). All ELISA plates were developed with ABTS and OD at 405 nm was read on a Biotech Instruments Microplate Autoreader. Anti-VV serum antibody titers were determined as endpoint titers 0.1 OD unit more than background (PBS/1% BSA) [23], [24]. BAL cytokine concentrations were interpolated from a protein standard curve. Detection limits were 125 pg/ml for IFNγ and IL-10.

## Results

### Poxvirus Clearance was Impaired in Animals with AAD

**Figure 1:**
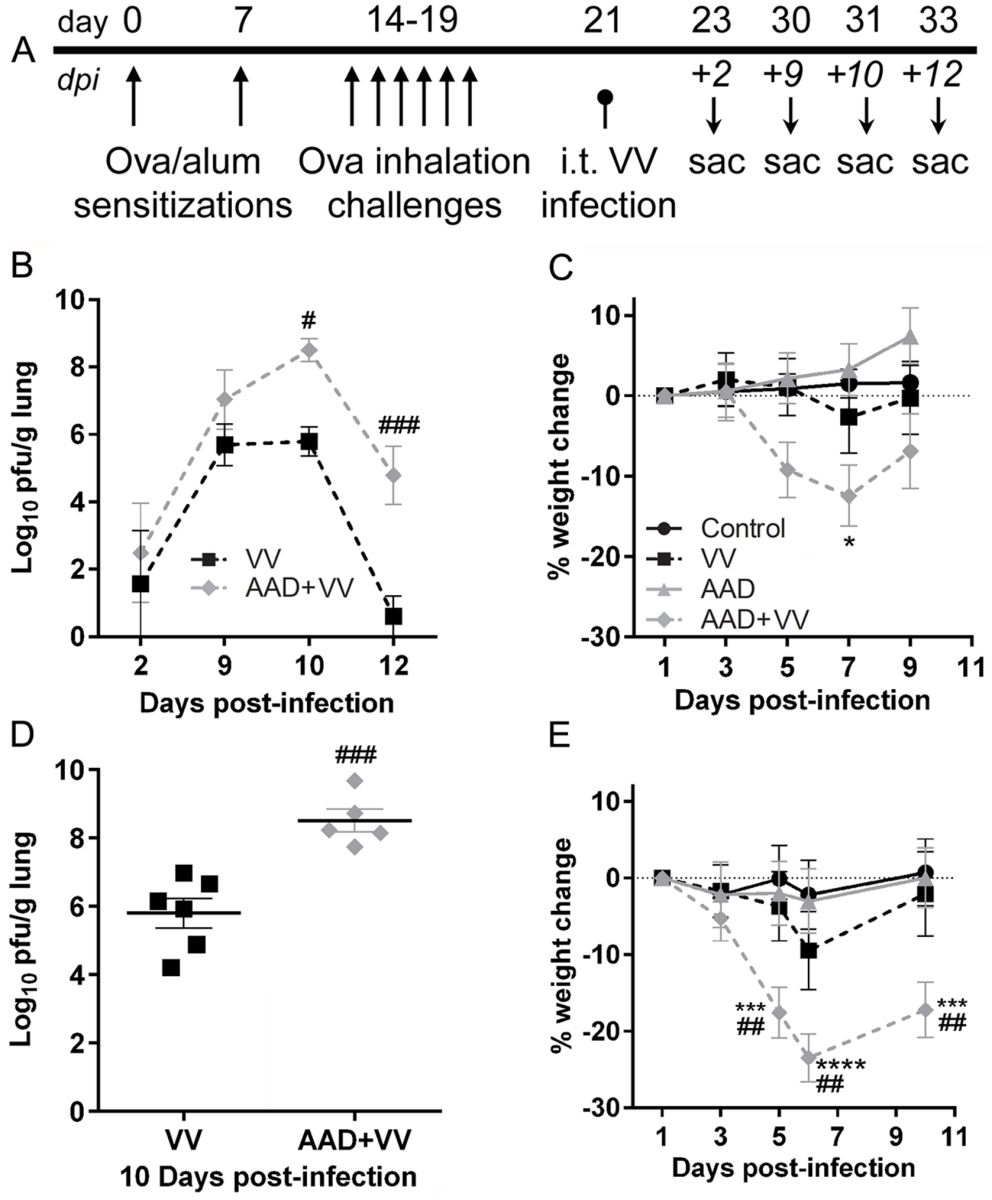
Preexisting AAD exacerbated pulmonary VV infection. (A) AAD was induced in mice by repeated OVA i.p. sensitizations and respiratory challenges over a course of 19 days. The resulting mice with AAD or control mice were inoculated at day 21 with 10^4^ PFU VV i.t. followed by monitoring for virus-induced pathology. VV titer and weight loss profiles from two separate cohorts of treated animals are shown. (B, D) Virus titers were measured in homogenized lung tissue using a viral plaque assay as described in the methods. Viral persistence and titer were significantly higher in AAD mice 10–12 dpi. The largest difference in virus titer between the VV mice and VV-infected AAD mice was observed at 10 dpi. (C, E) Mice were weighed starting one day after VV infection and the percent weight change was normalized to this day. The kinetics of weight loss after VV infection was not altered by AAD, but the maximal weight loss was significantly increased in AAD+VV mice. All values are represented as mean ± SEM, 4–14 mice per group. (D) Statistical significance was determined by a One-way ANOVA: ^###^ *P*<0.001 AAD+VV vs. VV. (B, C, E) Statistical significance was determined by a Two-way ANOVA with Bonferroni’s multiple comparisons test: *P<0.05, ***P<0.001, ****P<0.0001 AAD+VV vs. control; ^#^ *P*<0.05, ^##^ *P<0.01*, ^###^ *P*<0.001 AAD+VV vs. VV. The following abbreviations are used in all figure legends: VV, vaccinia virus; AAD, allergic airway disease; AAD+VV, allergic airway disease+vaccinia virus. The cohort of mice examined in panels B and C were also used in experiments shown in Figures 2–5. The cohort of mice examined in panels D and E were also used in experiments shown in Figures 6–7.

To induce allergic disease in the airways of animals, AAD mice were sensitized by i.p. injections of OVA/alum followed by repeated inhalation challenges with OVA (Fig. 1A). To investigate whether allergic inflammation in the lung compromises pulmonary immunity to VV, mice with or without AAD were inoculated with VV or mock-treated and sacrificed 2, 9, 10, or 12 dpi (Fig. 1A). Mice with AAD that were infected with VV had significantly higher lung virus titers compared to non-allergic mice 10–12 dpi (Fig. 1B, D). VV-infected mice with AAD lost weight sooner and had more severe weight loss, increased fur ruffling and lethargy compared to VV mice (Fig. 1C, E). These results suggest that mice with preexisting AAD have reduced poxvirus clearance and enhanced morbidity.

### Allergic Inflammation was Enhanced by a VV Infection

**Figure 2:**
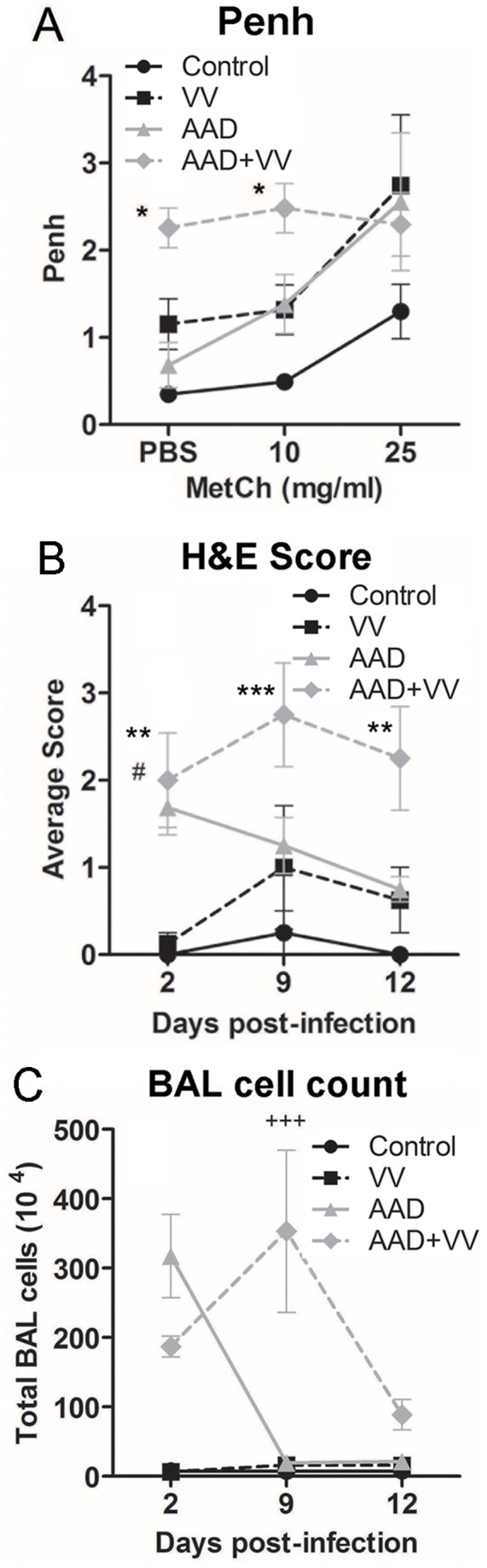
VV-infected AAD mice had increased signs of airway inflammation. (A) Non-invasive plethysmography was used to assess animal breathing. This analysis revealed increased responses to methacholine challenge (Penh) for control, VV and AAD mice at day 7 post-inoculation or mock treatment. In contrast, AAD mice inoculated with VV had elevated baseline Penh measurements suggesting an altered breathing pattern which was not sensitive to methacholine exposure. (B) Murine lung tissue was fixed in 10% formalin and paraffin-embedded sections were stained with H&E. Lung tissue inflammation was assessed by light microscopy and blindly scored using a semi-quantitative scale of 0–4, with a measure of 0 reflecting no inflammation, and 4 indicative of severe inflammation of peribronchiolar, periarterial and parenchymal spaces. AAD mice with or without VV infection had severe bronchiolar inflammation 2 dpi. VV-infected AAD mice had sustained inflammation through day 12 compared to AAD mice. (C) AAD mice with or without VV infection had elevated inflammatory cell infiltration in the BAL at 2 dpi. VV-infected mice with AAD had prolonged inflammatory cell infiltration in the BAL through 9 dpi. All values represented as mean ± SEM, 4–14 mice per group and representative of 3 independent experiments. Statistical significance was determined by a Two-way ANOVA with Bonferroni’s multiple comparisons test: *P<0.05, **P<0.01, ***P<0.001 AAD+VV vs. control; ^#^ *P*<0.05, AAD+VV vs. VV. ^+++^ *P*<0.001 AAD+VV vs. AAD.

Peribronchiolar inflammation is a predominant feature of AAD and is exaggerated during respiratory virus infections such as influenza and RSV [25]. Consistently, more persistent inflammation and alterations in animal respiration were associated with VV infection in the context of allergic lung disease. While the control, VV-inoculated and AAD mice had increased enhanced pause (Penh) in response to increasing doses of methacholine, this respiration pattern was perturbed in VV-infected AAD mice (Fig. 2A). Peribronchiolar inflammation was evident in mice with AAD, with or without VV, but absent in control mice or mice infected with VV alone by 2 dpi (Fig. 2B). Between days 9–12 post-infection, signs of peribronchiolar inflammation persisted in the VV-infected mice with AAD but were declining in the remaining treatment groups (Fig. 2B). At 2 dpi, total numbers of infiltrating cells in the BAL fluid were nearly 100-fold higher as a result of AAD (Fig. 2C). By 9 dpi, inflammatory cells in the BAL fluid from the mice with AAD had returned to the control levels, yet high numbers of infiltrating cells persisted in the lungs of VV-infected mice with AAD, coinciding with the peak of virus infection (Fig. 1B, Fig. 2C). In summary, alterations in breathing patterns, peribronchiolar inflammation and the accumulation of cells in alveolar spaces, were more severe and prolonged in VV-infected mice with AAD.

### Allergic Airway Disease Causes Disruption of Bronchial Epithelium, Perivascular Lymphoid Hyperplasia and Giant Cell Pneumonia

**Figure 3:**
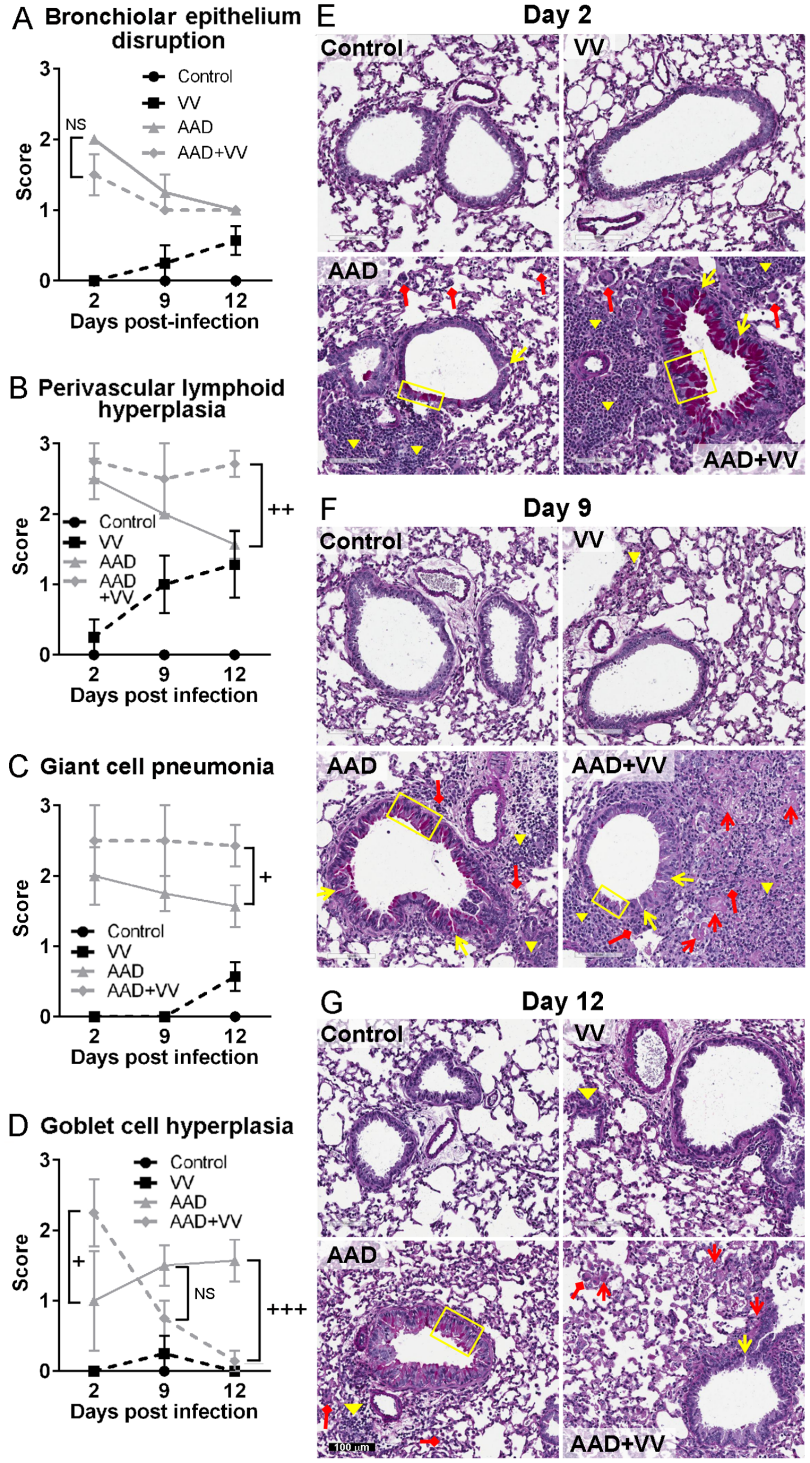
AAD caused increased epithelium disruption and cellular hyperplasia regardless of VV infection. Murine lung tissue was fixed in 10% formalin and paraffin-embedded sections were stained with PAS/hematoxylin and blindly scored for several pathophysiological parameters using a semi-quantitative scale of 0–3. Mice with AAD had (A) increased bronchiole epithelium disruption (yellow arrow) in the airways, (B) increased perivascular lymphoid hyperplasia (yellow inverted triangle), and (C) increased giant cell pneumonia (red diamond-headed arrow) at 2 and 9 dpi. (D) Goblet cell hyperplasia (yellow box) was significantly increased in AAD+VV mice at 2 dpi, but significantly decreased at 12 dpi compared to AAD mice. (E–G) PAS/hematoxylin-stained slides were digitally imaged with the Aperio Scan Scope CS system at 20× magnification. Multifocal necrotizing pneumonia is evident in the AAD+VV mice at 9 and 12 dpi as diffuse pink staining in the lung parenchyma (red arrow). Statistical significance was determined by a Two-way ANOVA with Bonferroni’s multiple comparisons test: NS - not significant, ^+^ *P*<0.05, ^++^ *P*<0.01 ^+++^ *P*<0.001 AAD+VV vs. AAD.

Disruptions in bronchiolar airway epithelium were observed in mice with AAD regardless of VV infection (Fig. 3A, E–G). Mice with AAD had increased levels of perivascular lymphoid hyperplasia and giant cell pneumonia which diminished over time. By contrast, the high levels of perivascular lymphoid hyperplasia and giant cell pneumonia observed in VV-inoculated mice with AAD were sustained during observations between days 9–12 (Fig. 3B–C, E–G). Interestingly, goblet cell hyperplasia was significantly increased at 2 dpi, but decreased at 12 dpi in the AAD+VV mice compared to AAD mice (Fig. 3D, E–G). Morphometric quantification of goblet cells was similar to pathological scoring (data not shown). Surprisingly, multifocal necrotizing pneumonia was evident in the AAD+VV mice 9–12 dpi (diffuse pink staining identified by red arrows) (Fig. 3F–G). By 12 dpi, 5/7 AAD+VV mice had developed multifocal necrotizing pneumonia compared to only 1/7 mice in the non-allergic, VV-infected group. There was no evidence of multifocal necrotizing pneumonia in the control or AAD mice during the time course (Fig. 3E–G). Therefore, VV infection in the context of AAD resulted in severe immunopathology that led to increased morbidity and the development of multifocal necrotizing pneumonia.

### Chemokine and Cytokine Expression in the Allergic Lung with Virus Infection

**Figure 4:**
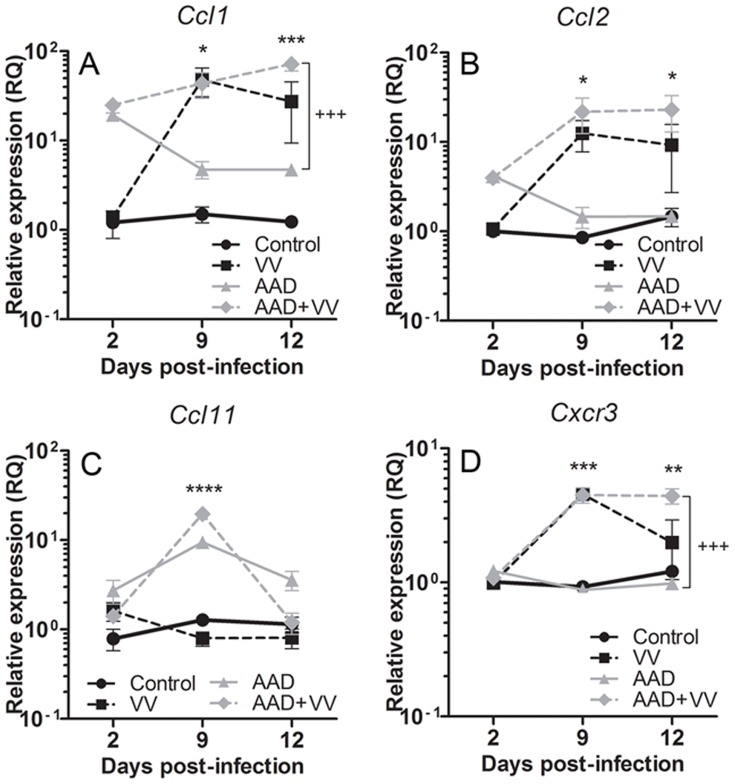
Induction of AAD and pulmonary VV inoculation altered the expression of chemokine ligand and receptor gene transcripts. Relative expression of gene transcripts in lung tissue was measured using qRTPCR. The expression of (A) *Ccl1*, (B) *Ccl2*, (C) *Ccl11* and (D) *Cxcr3* was significantly elevated in VV-infected AAD mice. Data are expressed as the mean relative expression ± SEM for four mice in each group and are representative of 2 independent experiments. Statistical significance was determined by a Two-way ANOVA with Bonferroni’s multiple comparisons test: *P<0.05, **P<0.01, ***P<0.001, ****P<0.0001 AAD+VV vs. control; ^+++^ *P*<0.001 AAD+VV vs. AAD.

**Figure 5:**
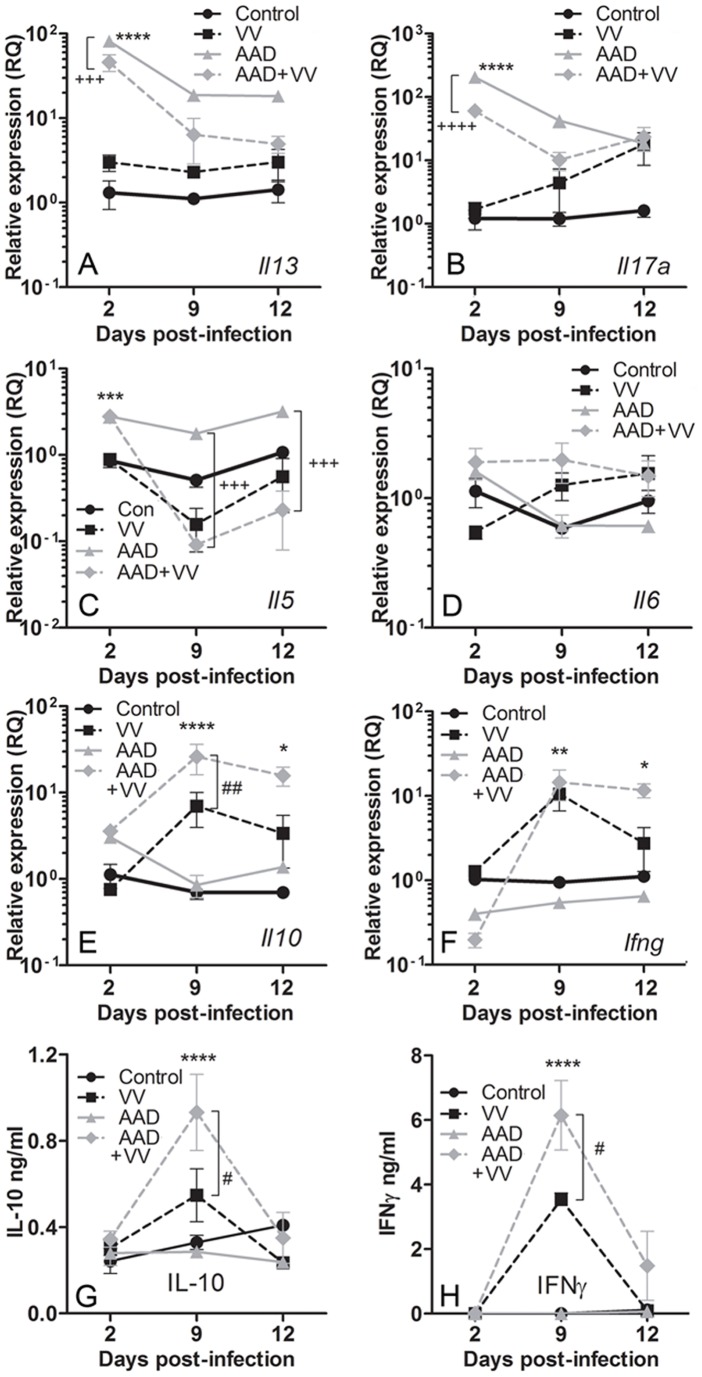
Induction of AAD and pulmonary VV inoculation altered expression of cytokines in the lungs. Relative expression of gene transcripts in lung tissue was measured using qRT-PCR. Transcripts for pro-allergic cytokines (A) *Il13*, (B) *Il17a* and (C) *Il5*, but not (D) *Il6*, were increased in AAD and VV-infected AAD mice. Transcripts for (E) *Il10* and (F) *Ifng* were elevated in VV-infected mice and VV-infected AAD mice. In VV-infected mice, (G) IL-10 and (H) IFNγ secretion in BAL fluid peaked by 9 dpi, as measured by ELISA. AAD mice inoculated with VV secreted more IL-10 and IFNγ at 9 dpi compared to non-allergic mice infected with VV. Data are expressed as the mean ± SEM for four mice in each group. Data are representative of 2 independent experiments. Statistical significance was determined by a Two-way ANOVA with Bonferroni’s multiple comparisons test: *P<0.05, **P<0.01, ***P<0.001, ****P<0.0001 AAD+VV vs. control; ^#^ *P*<0.05, ^##^ *P*<0.01 AAD+VV vs. VV; ^+++^ *P*<0.001, ^++++^ *P*<0.0001 AAD+VV vs. AAD.

The development of allergic inflammation requires a variety of mediators including chemokines and cytokines, the expression of which could polarize cellular infiltration or differentiation in the context of a VV infection. CCL1 and CCL2 induce the development and recruitment of inflammatory cells, particularly Th2 cells [26], [27]. Increased levels of chemokines in the lungs of VV-challenged mice have also been reported and may influence CD8^+^ T cell recruitment [13], [28]. Transcripts for *Ccl1* and *Ccl2* were initially elevated in the lungs of mice with AAD, and these transcripts decreased as lung inflammation resolved. However, VV-infected mice with AAD had sustained high levels of *Ccl1* and *Ccl2*, paralleling the induction of these chemokines in mice challenged with VV alone (Fig. 4A–B). By contrast, *Ccl11* (eotaxin 1) was significantly elevated at 9 dpi in lungs from mice with AAD regardless of VV exposure (Fig. 4C). Given the increased and persistent cellular infiltration, and sustained levels of chemokine transcripts in the lungs of VV-inoculated mice with AAD, the profile of cytokine expression in the lung during the course of infection was determined. Transcripts of pro-allergic cytokine genes *Il13*, *Il17* and *Il5* were significantly elevated in mice with AAD at early time points, but decreased by 9 dpi in contrast with *Il6* (Fig. 5A–D). The reduction in select pro-allergic cytokine transcripts was more pronounced in VV-infected mice with AAD and was concurrent with dramatic increases in *Il10* and *Ifng* transcript levels (Fig. 5E–F). VV inoculation of healthy mice also promoted pulmonary *Il10* and *Ifng* gene expression in the lungs, although this was less pronounced than in VV-infected mice with AAD (Fig. 5E–F). Additionally, significant quantities of secreted IL-10 and IFNγ were detectable in the BAL fluid of VV-infected mice with AAD at day 9, with much lower levels of these cytokines found in VV-infected mice without AAD (Fig. 5G–H). The chemokine receptor CXCR3 is highly expressed on Th1 cells and the expression of *Cxcr3* mimicked the pattern observed for *Ifng*, showing prolonged induction in VV-challenged mice with AAD (Fig. 4D). Increased *Il10* transcripts were not associated with increased infiltration of CD4^+^ T regulatory cells in the lungs, as *Foxp3* transcripts were only slightly attenuated by VV infection at 9 dpi, regardless of allergic inflammation, and numbers of CD4^+^ Foxp3^+^ T cells in the lung were unchanged (data not shown). These results suggest an attenuation of pulmonary allergic IL-13 and IL-17 production and enhancement of IL-10 and IFNγ synthesis in mice with allergic lung disease following VV infection.

### T cell Infiltration and Cytokine Production

**Figure 6:**
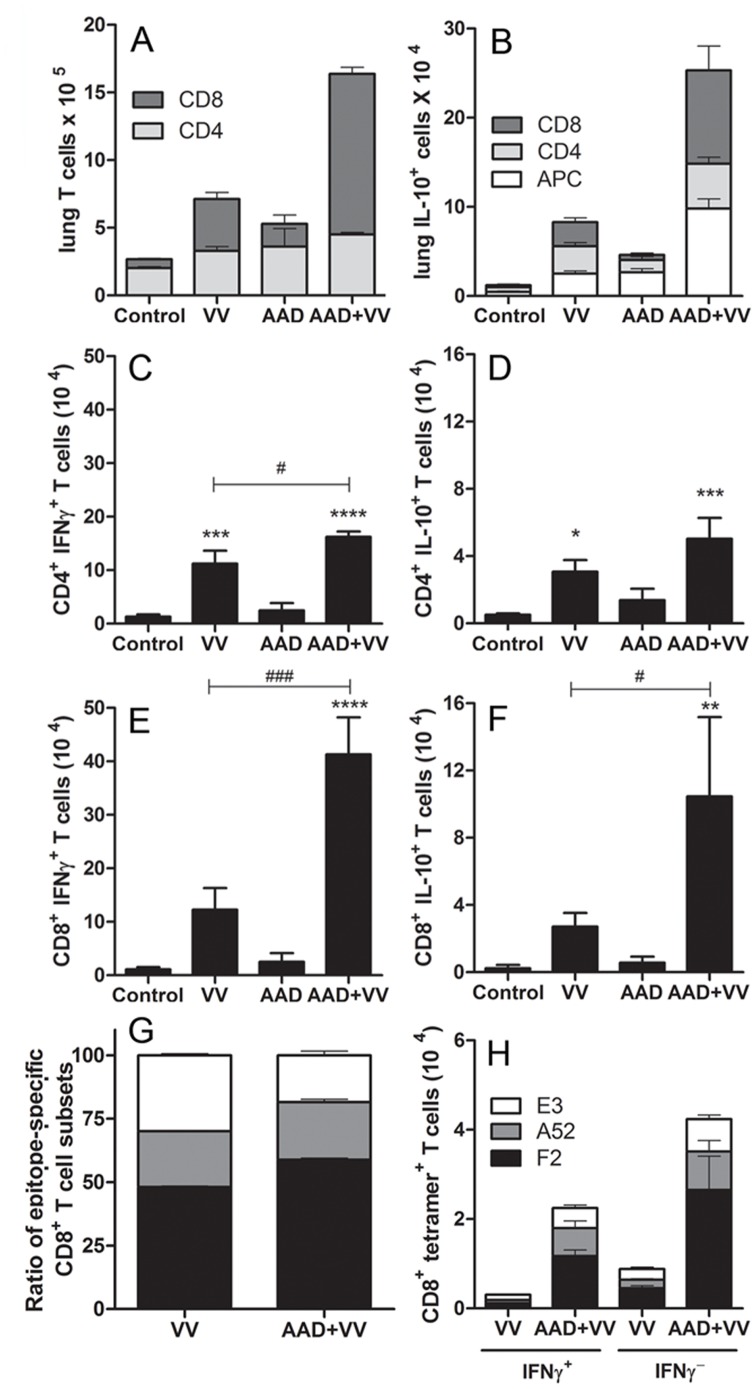
VV-infected AAD mice had increased CD8^+^ effector T cells in the lungs at 10 dpi. Lung tissue was harvested from mice 10 days after virus or mock inoculation. Single cell suspensions from individual animals were restimulated in vitro as described in the methods followed by antibody staining and flow cytometric analyses. The frequency of (A) CD4^+^ and CD8^+^, (B) IL-10^+^ (C) CD4^+^ IFNγ^+^, (D) CD4^+^ IL-10^+^, (E) CD8^+^ IFNγ^+^ and (F) CD8^+^ IL-10^+^ infiltrating lung T cells was determined by cell counts and FACS analysis using commercial antibodies as outlined in the methods. (G–H) Epitope-specific CD8^+^ T cells were determined by MHC I tetramer staining for H-2 class I epitopes (D^d^: E3 epitope; K^d^: A52 epitope; L^d^: F2 epitope) and FACS analysis (white bar E3, gray bar A52, black bar F2). Cells from dissociated lung tissue were restimulated in vitro and tetramer-stained as detailed in the methods. (G) Ratio of epitope-specific CD8^+^ T cells normalized to total CD8^+^ tetramer^+^ T cells. (H) Frequency of epitope-specific CD8^+^ T cells with or without IFNγ co-expression was determined. Statistical significance was determined by a One-way ANOVA with Bonferroni’s multiple comparisons test *P<0.05, **P<0.01, ***P<0.001, ****P<0.0001 AAD+VV vs. control; ^#^ *P*<0.05, ^###^ *P*<0.001 AAD+VV vs. VV. Results are expressed as mean ± SEM for (A–F) six mice in each group and are representative of 2 independent experiments or (G–H) 4–5 mice in each group.

Enhanced IL-10 production is observed in acute pulmonary virus infections, although the cellular source of this cytokine remains controversial and seems to vary with the pathogen and method of challenge [15], [29]–[32]. APC, including dendritic cells and macrophages, were suggested as potential sources of IL-10 after cowpox infection [15]. Thus, inflammatory pulmonary infiltrates were examined at 10 dpi (peak of VV titer) to identify the source of secreted IL-10 and IFNγ. The frequency of resident CD4^+^ T cells increased slightly in mice with AAD independent of VV inoculation (Fig. 6A). Interestingly, a VV infection significantly increased the number of pulmonary CD8^+^ T cells by nearly 6-fold in non-allergic mice (*P*<0.01 compared to control) and a remarkable 18-fold in AAD mice (*P*<0.0001 compared to control) (Fig. 6A). VV-infected mice produced similar numbers of APC, CD4^+^ and CD8^+^ T cells expressing IL-10 (Fig. 6B). The frequency of CD4^+^ IFNγ^+^, CD4^+^ IL-10^+^, CD8^+^ IFNγ^+^ and CD8^+^ IL-10^+^ T cells increased following VV infection and pulmonary infiltration of these cells was further amplified in VV-infected mice with AAD (Fig. 6C–F). Approximately 80% of the IL-10^+^ T effector cells also co-expressed IFNγ (data not shown). Interestingly, VV-infected mice with AAD had an increased frequency of IL-10^+^ cells from all subsets, but the majority of IL-10-producing cells were APC or CD8^+^ T cells (Fig. 6B). Analyses using specific viral peptides bound to MHC class I-restricted tetramers demonstrated qualitative and quantitative changes in CD8^+^ T cell responses upon infection of mice with or without AAD. Increased numbers and percentage of CD8^+^ T cells recognizing an immunodominant epitope from the viral F2 antigen in the context of H-2 L^d^ were observed in mice with AAD compared with mice challenged with VV alone (Fig. 6G–H). By contrast, CD8^+^ T cell responses to H-2 D^d^ complexed with an epitope from the viral E3 antigen contributed a lower proportion of total virus-specific T cells in mice with AAD than in mice challenged with VV alone (Fig. 6G). Approximately 1/3 of these tetramer^+^ CD8^+^ T cells expressed significant amounts of IFNγ in virus infected mice with or without AAD (Fig. 6H). Together, these studies demonstrate a qualitative and quantitative change in CD8^+^ T cell responses to VV immunodominant epitopes in mice with AAD.

### VV-specific Immunoglobulins

**Figure 7:**
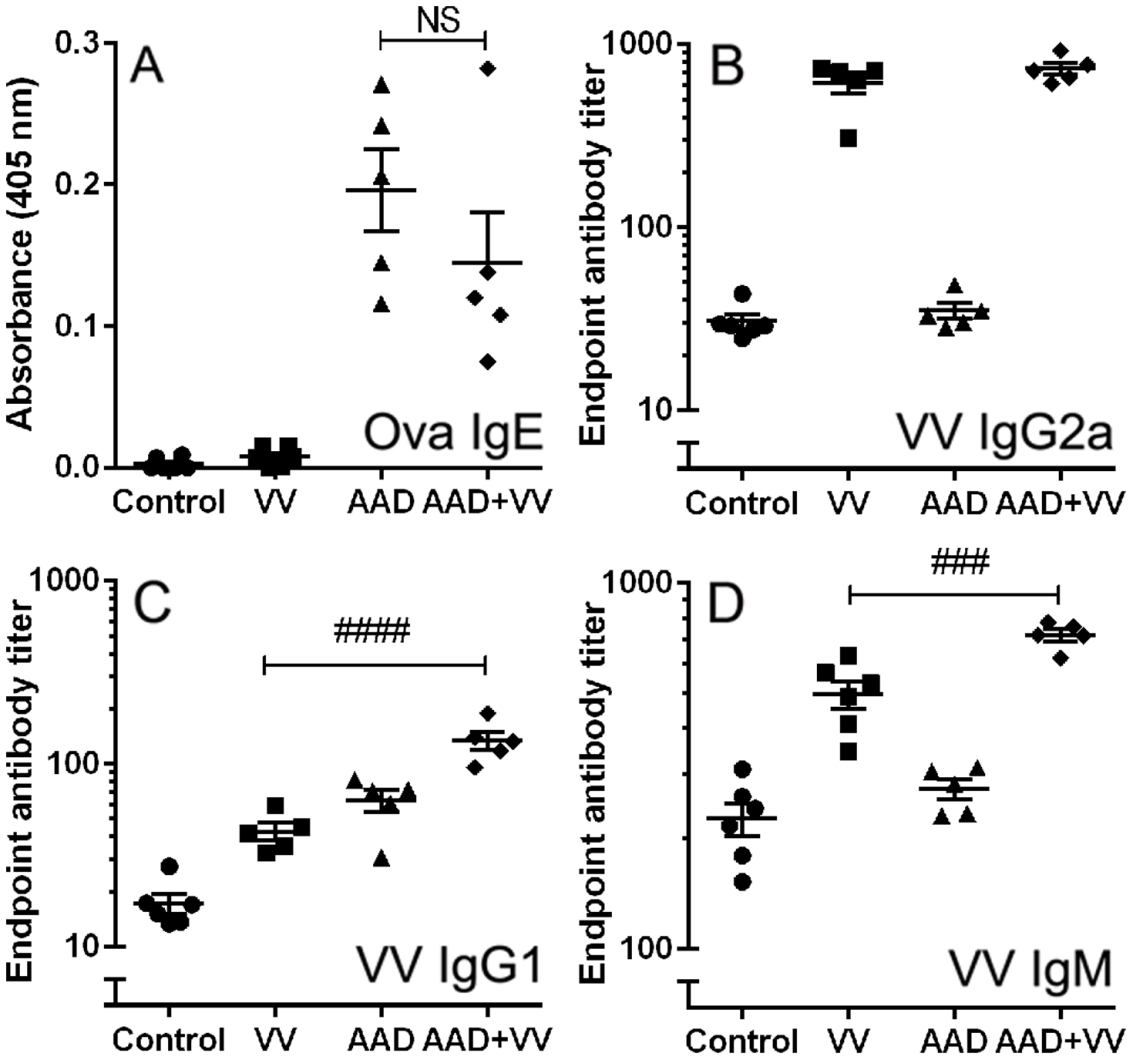
Titers of VV-specific IgG1 and IgM were increased in VV-infected AAD mice at 10 dpi. Serum levels of (A) OVA-specific IgE, (B) VV-specific IgG2a, (C) VV-specific IgG1 and (D) VV-specific IgM were determined by ELISA. Serum antibody levels were determined by antibody capture on OVA or virus coated plates with detection of bound antibodies via enzyme linked secondary reagents as outlined in the methods. (A) OVA-specific IgE levels from mouse serum are reported as absorbance values. (B–D) VV-specific antibody titers were determined as endpoint titers 0.1 OD unit more than background (PBS/1% BSA). Statistical significance was determined by a One-way ANOVA with Bonferroni’s multiple comparisons test: NS – not significant; ^###^ *P*<0.001, ^####^ *P*<0.0001 AAD+VV vs. VV. Results are expressed as the mean ± SEM for 5–6 mice in each group and are representative of 3 independent experiments.

Despite the increased frequency of CD8^+^ IFNγ^+^ T cells, VV-infected mice with AAD were substantially impaired in their ability to control VV replication, as evidenced by pulmonary VV titers that were nearly 500 times greater than non-allergic VV-infected mice (Fig. 1B, D). We hypothesized that alterations in early VV-specific antibody responses may also influence the susceptibility of AAD mice to a VV infection. As expected, mice with AAD sensitized with OVA had high levels of OVA-specific IgE that were not altered by a VV infection (Fig. 7A). Anti-VV IgG2a was detected by 10 dpi and the IgG2a response was not altered by AAD (Fig. 7B). In contrast, VV-infected mice with AAD developed significantly greater serum levels of anti-VV IgG1 and anti-VV IgM compared to VV-infected mice without AAD (Fig. 7C–D). Therefore, the levels of distinct subclasses of anti-viral antibodies produced were significantly altered in mice with AAD.

### Blocking IL-10 Signaling Perturbed IFNγ Secretion in the Lungs without Altering Animal Weight Loss or Virus Titer

**Figure 8:**
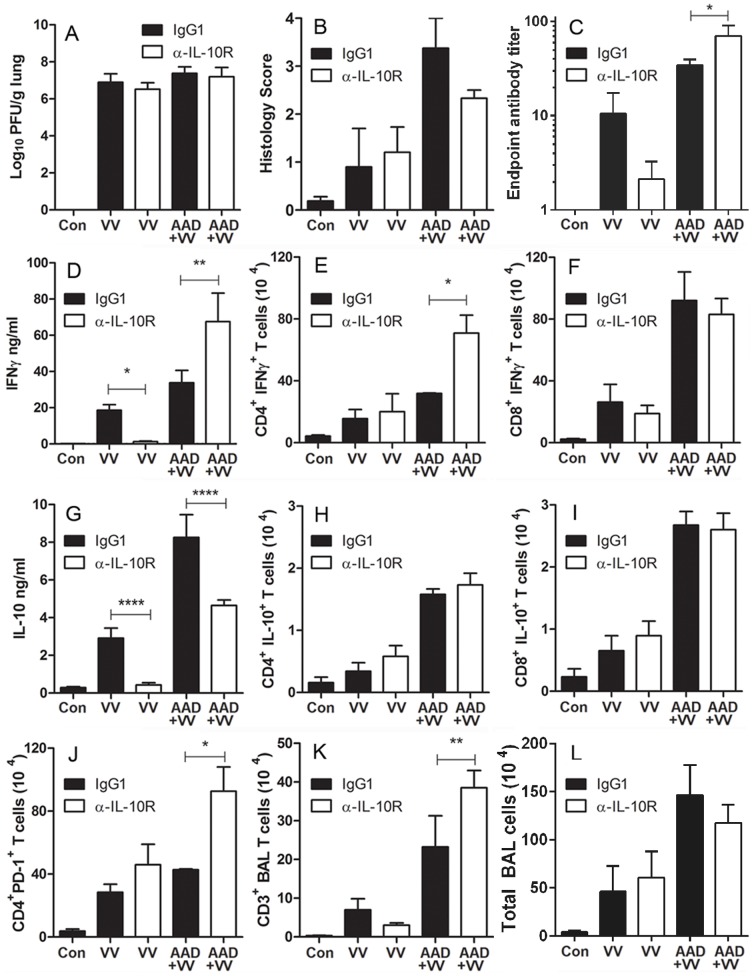
Blocking IL-10R signaling in VV-infected AAD mice resulted in altered disease severity. VV-infected mice were treated with Rat IgG1 control mAb or αIL-10R blocking mAb at 3 (i.p.), 4 (i.n.) and 6 dpi (i.p.), and these animals were sacrificed on day 9. (A) Lung VV titers and (B) bronchiole inflammation were not altered by IL-10R mAb blockade. Blocking IL-10R signaling increased (C) levels of VV-specific IgG1 in serum, (D) IFNγ protein levels in BAL fluid and (E) the frequency of infiltrating CD4^+^ IFNγ^+^ T cells in the lungs of VV-infected AAD mice. Treatment with an αIL-10R mAb did not alter the infiltration of (F) CD8^+^ IFNγ^+^, (H) CD4^+^ IL-10^+^ or (I) CD8^+^ IL-10^+^ T cells, butincreased recruitment of (J) CD4^+^ PD-1^+^ T cells in the lungs of VV-infected AAD mice. Blocking IL-10R significantly decreased (G) BAL IL-10 protein secretion and significantly increased (K) BAL T cells but not (L) total BAL cells in VV-infected mice with AAD. Statistical significance was determined by a One-way ANOVA with Bonferroni’s multiple comparisons test: *P<0.05, **P<0.01, ****P<0.0001 αIL-10R mAb vs. IgG1. Results are expressed as the mean ± SEM for 3–5 mice in each group and are representative of 2 independent experiments.

In a pulmonary influenza virus infection, IL-10 has been reported to positively and negatively regulate virus-induced pathology [29], [30]. Upon a cowpox virus challenge, disruption of IL-10 expression in mice resulted in increased inflammatory infiltrates in the lungs of mice and unexpectedly, increased susceptibility to re-infection [15]. To determine the role of secreted IL-10 in VV-infected mice with AAD, the animals were treated with a blocking anti-IL-10R (αIL-10R) mAb. Administration of the αIL-10R mAb in vivo did not substantially alter pulmonary virus titer, lung histopathology, weight loss or Penh in mice challenged with VV in the presence or absence of AAD (Fig. 8A–B; data not shown). However, in VV-infected mice with AAD, treatment with αIL-10R mAb significantly increased serum titers of anti-VV IgG1 (Fig. 8C). In contrast, IL-10R blockade in VV-infected mice without AAD resulted in a decrease in VV-specific IgG1 (Fig. 8C). Blocking IL-10 signaling enhanced the secretion of BAL fluid IFNγ levels in the lungs of AAD mice inoculated with VV, in contrast with reduced production of this cytokine in mice exposed to VV only (Fig. 8D). Notably, IL-10R blockade in AAD mice infected with VV increased the frequency of CD4^+^ IFNγ^+^ (Fig. 8E), but not CD8^+^ IFNγ^+^ T effector cells in the lungs (Fig. 8F). This suggests the increased secretion of IFNγ in BAL fluid from VV-infected AAD mice treated with αIL-10R mAb is likely due to enhanced recruitment of CD4^+^ T cells. Treatment with a blocking IL-10R mAb decreased the secretion of IL-10 without altering the number of IL-10^+^ T cells in the lungs (Fig. 8G–I). Additionally, IL-10R blockade increased the expression of the inhibitory molecule PD-1 on CD4^+^ T cells in the lungs of VV-infected mice with AAD (Fig. 8J). Moreover, the number of airway T cells also increased in VV-infected mice with AAD following IL-10R blockade whereas total numbers of BAL cells remained unchanged (Fig. 8K–L). This was in contrast to results observed following disruption of IL-10 signaling in mice inoculated with VV alone. These results suggest IL-10 plays a role in modulating CD4^+^ T cell recruitment and activation as well as the induction of virus-specific antibodies in response to a VV infection in a pro-allergic environment.

## Discussion

Respiratory viruses can induce or worsen pulmonary inflammation and tissue damage associated with allergic lung disease, with such changes being driven in part by the viral pathogen [1], [2]. Poxviruses such as VV are readily transmitted via respiratory routes, and importantly, these viruses encode many potent gene products which promote immune evasion and virus spread [33]. IL-10 is known to suppress inflammation and associated tissue damage as well as lead to chronic or more persistent virus infections. As shown here, IL-10 was produced by a variety of APC and T cells during respiratory VV infection, and disruption of IL-10 signaling in these animals altered T and B cell function. Mice with established atopic lung disease were significantly affected by respiratory VV infection with severe peribronchiolar inflammation, weight loss and diminished viral clearance culminating in the development of multifocal necrotizing pneumonia.

Histopathologic examination of lung tissue from both groups of AAD mice revealed the classical features of allergic inflammation including epithelial damage, perivascular lymphoid and goblet cell hyperplasia and multinucleated giant cells (MNG) [34], [35]. The VV infection in the context of AAD caused prolonged accumulation of perivascular lymphoid and giant cells, in contrast to mice with AAD only. MNG cells were also detected in non-allergic, VV-infected mice at 12 dpi, consistent with reports of giant cell pneumonia in the lungs of patients infected with severe measles or respiratory syncytial virus infections [36]–[38]. Compared to AAD mice, AAD+VV mice had attenuated scores for goblet cell hyperplasia, which is consistent with the relative decrease in Th2 gene transcripts.

Very high levels of pulmonary IL-10 were detected along with the infiltration of CD8^+^ and CD4^+^ T cells as well as alterations in anti-viral serum antibodies in VV challenged mice with AAD. VV-infected mice with AAD had altered breathing patterns as measured by greater baseline Penh, which could be due to increased atelectasis resulting from constriction of the airways and increased mucus production blocking the airways. Indeed, the latter was readily apparent, especially at 2 dpi. Increased atelectasis might also limit the methacholine responsiveness by limiting the proportion of the lung responding to challenge. However, Penh does not directly measure airway reactivity, and some of the observed differences from control groups could be due to altered tachypnea (rate of respiration) or the combination of AAD and VV infection affecting CNS control of respiratory function [39]. Importantly, the increased Penh measurements correlated well with all other parameters of inflammation.

### Induction of T cell-derived Cytokines during a Pulmonary Poxvirus Infection

Virus transmission and pathogenesis are modulated in part by localized secretion of T cell-derived IFNγ, IL-17 and Th2-associated cytokines. Healthy mice inoculated with VV displayed enhanced pulmonary recruitment of CD4^+^ and CD8^+^ T cells producing IFNγ and IL-17, consistent with reports that these cells promote viral clearance [14], [40], [41]. Remarkably, despite increased numbers of lung infiltrating CD8^+^ IFNγ^+^ T cells and increased secretion of BAL IFNγ, VV titers were significantly higher in VV-infected mice with AAD compared to VV-infected non-allergic mice. By contrast, Barends and colleagues reported that although pulmonary inoculation of RSV and influenza virus induced high IFNγ production, IFNγ levels were similar between AAD and control mice [3]. While an RSV infection of allergic animals results in enhanced Th2 cytokine production in the lungs, influenza respiratory challenge of AAD mice reduced lung IL-4, IL-5, and IL-13 expression [3]. Here, pulmonary VV inoculation mirrored influenza infection as pulmonary transcripts for IL-5, IL-13, and IL-17 were significantly decreased in VV-infected mice with AAD.

### Sources of Pulmonary IL-10 and Changes in Virus-specific T cells

In vitro studies have suggested that macrophages and dendritic cells are potential sources of IL-10 following cowpox virus or VV infection [15], [42]. However, production of pulmonary IL-10 following a respiratory influenza A virus or RSV infection is driven by T cells, although reports conflict as to whether CD4^+^ or CD8^+^ T cells are the predominant source of this cytokine [29]–[32]. In this study, IL-10^+^ lung resident APCs, CD4^+^ T cells and CD8^+^ T cells were detected in equal numbers following a VV infection of control mice. VV-inoculation of mice with AAD resulted in an increase in IL-10^+^ APCs and CD8^+^ IL-10^+^ T cells, whereas numbers of infiltrating CD4^+^ IL-10^+^ T cells were relatively unchanged compared to VV-infected control mice. Among APC, IL-10^+^ macrophages predominated at late stages of VV infection in mice with AAD. T regulatory cells were not likely responsible for IL-10 production in response to VV as less than 10% of CD8^+^ IL-10^+^ cells and fewer than 2% of CD4^+^ IL-10^+^ T cells co-expressed Foxp3 (data not shown). This is in stark contrast to an RSV infection, where a significant number of the recruited CD4^+^ IL-10^+^ T cells co-express Foxp3 [32].

A shift in the hierarchy of CD8^+^ T cell responses to immunodominant viral epitopes F2, A52 and E3 was apparent in mice with AAD compared to control mice challenged with virus. A shift in the hierarchy of CD8^+^ T cell responses to these epitopes has been reported to be dependent on the route of immunization [43]. For example, with i.p. virus inoculation, nearly 20–25% of the CD8^+^ IFNγ^+^ T cell response is directed against these viral epitopes and the epitope hierarchy is F2>A52>E3 [43], [44]. Furthermore, vaccination against poxviruses is most effective via skin scarification; using this route of inoculation, 40% of the virus-specific CD8^+^ T cells elicited in mice responded to these three viral epitopes and follow a F2>E3>A52 hierarchy [44]. Using respiratory VV infection, a natural route of transmission, nearly 60% of the pulmonary CD8^+^ IFNγ^+^ T cells recognized these immunodominant epitopes. Interestingly, the hierarchy of CD8^+^ T cell responses following pulmonary infection in mice with AAD mirrors that observed following i.p. infection. This shift may reflect the more systemic spread of the virus in animals with AAD, or the influence of the allergic lung microenvironment on T cell-APC interactions. These findings have important implications in terms of the specificity of anti-viral immune responses in allergic individuals.

### Altered Antibody Responses to Virus in Mice with AAD

In mice, IFNγ-producing Th1 cells provide help to IgG2a-secreting B cells [40], [45]. IgG2a was the most abundant anti-VV antibody isotype detected, yet serum levels of anti-VV IgG2a were similar in the VV-infected mice with or without AAD despite significantly higher virus load, BAL IFNγ levels, and numbers of resident lung tissue CD8^+^ IFNγ^+^ cells in the VV-infected AAD mice. Remarkably, titers for IgG1 and IgM anti-VV antibodies were significantly increased in VV-infected AAD mice compared to VV-infected control mice. Despite this increase in IgG1 and IgM, virus spread and titers were consistently higher in the allergic mice, suggesting impaired immunity. Like IgE, antigen-specific IgG1 is elicited during allergic diseases and parasite infections. The observed Ig class switching of virus-specific antibodies detected in animals with AAD may have implications in terms of pathogen-specific humoral immunity in the context of the allergic lung. A similar shift towards Th2-associated isotypes has been reported in aeroallergen-sensitive adults and children with enhanced development of IgG4 antibodies (analogous to murine IgG1), specific for the pulmonary bacterial pathogen *Haemophilus influenza* [46]. Furthermore, infants who developed dust mite allergies displayed decreased titers of human IgG1 (analogous to murine IgG2a) specific for several pulmonary bacterial pathogens, further supporting a role for atopic diseases in biasing humoral immunity [46]. Alterations in circulating antibody isotypes may reflect not only deficits in the immune responses of asthmatic individuals to bacteria, but also viral pathogens. Thus, the skewing of anti-VV antibody isotypes in mice with AAD may reflect an important bias mediated by the allergic lung microenvironment.

### IL-10-mediated Suppression of Pulmonary Inflammation in Virus-infected Mice with AAD

Previous studies have suggested IL-10 may play a role in virus clearance [47]. Nonetheless, here and in studies of cowpox virus, respiratory poxvirus clearance was not dependent on IL-10, but pulmonary IL-10 signaling influenced inflammation and the development of host immunity [15], [42]. Protective effects of IL-10 have been proposed in pulmonary influenza or RSV infections, where IL-10R blockade results in increased animal weight loss, greater AHR and enhanced pulmonary IFNγ production, despite unchanged viral titers [30]–[32]. Interestingly, disrupting IL-10 signaling did not change the number of pulmonary infiltrating IL-10^+^ CD4^+^ or CD8^+^ T cells, but did result in decreased secretion of IL-10 into the airway. Although this result was unexpected, previous studies have shown that IL-10 can act in a positive autocrine factor directly on T cells [48]. Additionally, IL-10 acts in an autocrine manner during other models of pulmonary virus infection [7], [31]. Blocking IL-10R signaling did not alter lung VV titers, histopathology or Penh, but enhanced recruitment of CD4^+^ T cells expressing IFNγ or PD-1. This finding is consistent with studies of an acute LCMV infection where IL-10 suppressed CD4^+^ but not CD8^+^ T cell function [49]. PD-1 expression on T lymphocytes is typically associated with a chronic virus infection and T cell exhaustion, as well as regulated IL-10 production [50], [51]. Blockade of PD-L1 increased CD8^+^ T cell antiviral cytokine production and decreased the viral burden in RSV-infected APCs [52].

### Pulmonary Immunity to Poxvirus Infection and Allergic Disease

The WHO estimates nearly 235 million individuals suffer from AAD such as asthma with an increasing incidence of AAD in developing countries. Respiratory viral infections such as RSV and influenza are known to exacerbate pulmonary inflammation associated with AAD [53], [54]. In the current study, VV-infected mice with AAD had increased virus replication, pulmonary inflammation, weight loss, and signs of multi-focal necrotizing pneumonia, demonstrating the deleterious consequences of pulmonary poxvirus transmission in individuals with respiratory allergic diseases. While CD8^+^ T cell responses and IFNγ production increased after VV inoculation of mice with AAD, so too did pulmonary IL-10 production. This, along with a shift in the hierarchy of CD8^+^ T cell epitopes recognized by mice with AAD and skewed anti-VV antibody production, may contribute to dysregulated host immunity to VV in individuals with AAD. With the increasing incidence of allergic disease, understanding how an atopic environment within the lungs shapes the specificity of T and B cell responses will be critical to limiting lung inflammation and pathology resulting from pulmonary virus infections.
